# Combined Effect of Poly(lactic acid)-Grafted Maleic Anhydride Compatibilizer and Halloysite Nanotubes on Morphology and Properties of Polylactide/Poly(3-hydroxybutyrate-co-3-hydroxyhexanoate) Blends

**DOI:** 10.3390/ma16196438

**Published:** 2023-09-27

**Authors:** Nawel Mokrane, Mustapha Kaci, José-Marie Lopez-Cuesta, Nadjet Dehouche

**Affiliations:** 1 Laboratoire des Matériaux Polymères Avancés, Faculté de Technologie, Université de Bejaia, Béjaïa 06000, Algeria; nawel.mokrane@univ-bejaia.dz (N.M.); mustapha.kaci@univ-bejaia.dz (M.K.); nadjet.dehouche@univ-bejaia.dz (N.D.); 2 Polymères Composites et Hybrides (PCH), IMT Mines Ales, 6, Avenue de Clavières, 30319 Alès, France

**Keywords:** PLA, PHBHHx, polymer blends, halloysite nanotubes, PLA-*g*-MA compatibilizer, morphology

## Abstract

Given the global challenge of plastic pollution, the development of new bioplastics to replace conventional polymers has become a priority. It is therefore essential to achieve a balance in the performances of biopolymers in order to improve their commercial availability. In this topic, this study aims to investigate the morphology and properties of poly(lactic acid) (PLA)/ poly(3-hydroxybutyrate-co-3-hydroxyhexanoate) (PHBHHx) (at a ratio of 75/25 (*w*/*w*)) blends reinforced with halloysite nanotubes (HNTs) and compatibilized with poly(lactic acid)-grafted maleic anhydride (PLA-*g*-MA). HNTs and PLA-*g*-MA were added to the polymer blend at 5 and 10 wt.%, respectively, and everything was processed via melt compounding. A scanning electron microscopy (SEM) analysis shows that HNTs are preferentially localized in PHBHHx nodules rather than in the PLA matrix due to its higher wettability. When HNTs are combined with PLA-*g*-MA, a finer and a more homogeneous morphology is observed, resulting in a reduction in the size of PHBHHx nodules. The presence of HNTs in the polymer blend improves the impact strength from 12.7 to 20.9 kJ/mm^2^. Further, with the addition of PLA-*g*-MA to PLA/PHBHHX/HNT nanocomposites, the tensile strength, elongation at break, and impact strength all improve significantly, rising from roughly 42 MPa, 14.5%, and 20.9 kJ/mm^2^ to nearly 46 MPa, 18.2%, and 31.2 kJ/mm^2^, respectively. This is consistent with the data obtained via dynamic mechanical analysis (DMA). The thermal stability of the compatibilized blend reinforced with HNTs is also improved compared to the non-compatibilized one. Overall, this study highlights the effectiveness of combining HNTs and PLA-*g*-AM for the properties enhancement of PLA/PHBHHx blends.

## 1. Introduction

The massive use of petroleum-based non-biodegradable polymers has led to serious environmental pollution due to the dissemination of plastics waste. To mitigate this propensity, scientists are committed to develop and synthesize biobased/biodegradable polymers with eco-friendly characteristics [1,2]. Within this new class of biomaterials, PLA is gaining attraction in the market, and it represents one of the most commercialized bio-based polymers capable of substituting some petroleum-based plastics [3]. PLA is a semi-crystalline polymer with a crystallinity content of usually less than 10% due to its low crystallization rate. PLA is a biodegradable polyester derived from renewable materials like sugarcane or corn [4]. Because of its advantageous properties, including strength, non-toxicity, biocompatibility, and transparency, PLA is used in a variety of industrial fields, involving particularly the medical and packaging sectors [5]. Nevertheless, some drawbacks of PLA include brittleness, poor toughness, slow crystallization, and poor barrier properties, which cannot challenge regular commodity polymers [6,7,8]. Taking into account these weak points, significant progress in the field of PLA modification has been made, as seen by the growing number of scientific articles dedicated to this research subject [9,10]. One of the solutions recommended to address these limitations is to blend PLA with other flexible polymers to improve the blend properties, besides its economic and technical viability. In this regard, the literature [11] indicates that PLA has been blended with several biopolymers to enhance both ductility and processability, including poly((butylene adipate)-co-terephthalate) (PBAT), poly((butylene succinate)-co-adipate) (PBSA), polybutylene succinate (PBS), and polycaprolactone (PCL). Recently, polyhydroxyalcanoates (PHAs) have attracted the interest of both academia and the industry, especially for packaging purposes, where the demand is increasing despite the high production cost, which still remains the main challenge for extending the use of PHAs to wider industrial applications [12]. However, from the perspective of environmental protection, PHAs are a perfect fit for markets in which biodegradability in various environmental conditions is essential. Among the PHA family, the most well known are polyhydroxybutyrate (PHB) and the copolymer poly(3-hydroxybutyrate-co-3-hydroxyvalerate) (PHBV) [12].

Poly(3-hydroxybutyrate-co-3-hydroxyhexanoate) (PHBHHx) is characterized by a lower crystallinity and a wider processing window than the previous PHAs. Further, PHBHHx is soft with a high elongation at break and a relatively low tensile strength and modulus. Blending PHBHHx with PLA could be an effective way of improving the toughness and flexibility of PLA-based blends because of their complementary mechanical properties [13,14]. In this regard, the literature [15,16,17] reveals a significant enhancement of the mechanical properties in blends of PLA/PHBHHx at 80/20 wt.%, whereas an increase in toughness is observed at 10 wt% of PHBHHx, despite the fact that PLA and PHBHHx are not thermodynamically miscible [18]. The morphology and properties of PLA/PHBHHx blends were studied by Lim et al. [17]. The authors reported that as the amount of PHBHHx increases in the blend, a drop in the PLA crystallization is noted. They concluded that the addition of a small amount of PHBHHx to PLA is sufficient to improve the ductility and toughness of PLA/PHBHHx blends [17].

The literature [19,20,21] also reports that the addition of nanofillers, such as nanosilica particles, montmorillonite, graphene oxide, carbon nanotubes, and others, to immiscible polymer blends may improve interfacial adhesion and, subsequently, blend compatibility. The form, specific surface area, surface chemistry, and distribution of nanofillers in polymer blends all have a substantial influence on their property enhancement [22]. The interfacial interactions between particles and the polymer matrix are directly affected by wetting parameters such as the surface tension, viscosity, and interfacial energy. These parameters determine the degree of wetting or spreading of particles on polymer surfaces, thus influencing their dispersion and localization [23]. Nanofillers frequently cluster within the phase that has the least interfacial tension. When nanofillers are located in the dispersed phase, the droplet size increases due to the increase in the viscoelasticity of the phase [24]. The size of the dispersed phase decreases as the nanoparticles are concentrated in the matrix [25]. The selective localization of the nanofillers at the interface results in a finer droplet morphology and minimizes droplet coalescence during processing, thus promoting the compatibilizing effect of nanofillers [26]. In fact, there are many papers dealing with the structure–property relationships of PLA-based blends and organo-modified layered silicate (OMLS) [27,28,29,30]. Among these, Salehiyan et al. [28] investigated the rheological properties of ternary blends based on PLA/PCL/mLLDPE (metallocene-catalyzed linear low-density polyethylene) at a ratio of 70/30/10 and at 1, 2, and 4 phr of OMLS. The authors reported that the mineral filler acts as a compatibilizer between mLLDPE and the PLA/PCL blend, particularly at a higher content, reducing the size of PCL droplets. Nofar et al. [31] studied the effect of organo-modified MMT on the morphology of PLA/PBAT (75/25) blends. The authors reported that OMLS is located in the interface between the two polymer phases, as predicted by thermodynamics laws. Further, it was also found that an increase in the clay level from 1 to 5 wt.% results in an excess of OMLS being localized in the PLA matrix, which is due to a stronger thermodynamic affinity of the clay for PLA than for PBAT. Nuzzo et al. [32] studied the rheological characteristics of PLA/PA11 blends at different loading rates using organo-modified montmorillonite, sepiolite, and multi-walled carbon nanotubes. The authors observed that all three types of nanoparticles are able to induce the switch from drop matrix to co-continuous morphology. Further, the nanoparticles are preferentially located in the dispersion regions of the minor phase of PA11. At a critical level, the nanoparticles contained in the minor phase modify the morphology of PLA/PA11 droplets into a stable co-continuous morphology.

Among mineral fillers, halloysite nanotubes (HNTs) are another promising class of mineral clays with the molecular formula of Al_2_Si_2_O_5_(OH)_4_.H_2_O. They are used as fillers and reinforcements in polymers as a cheap alternative to expensive carbon nanotubes [33]. Indeed, the biocompatibility, high aspect ratio, thermal stability, high strength, and availability at a low cost of HNTs are the main advantages in nanocomposite material applications [34]. Moreover, the ability of HNTs to improve the compatibility between immiscible polymer blends has also been reported [35]. In this regard, Pal et al. [35] indicated that the surface modification of halloysite nanotubes by N-(β-aminoethyl)-ɣ-aminopropyl -trimethoxysilane improves the compatibility between polyoxymethylene (POM) and polypropylene (PP) blends. Indeed, the domain size of POM was reduced after adding 1 wt.% of modified-HNTs, leading to the conclusion that modified HNTs improve the interfacial interactions between PP and POM. Mishra et al. [36] also observed an improved interfacial adhesion between nanocomposite blends based on polyetherimide/silicone rubber in the presence of halloysite nanotubes used as the compatibilizer. Moreover, the thermal and mechanical properties of the nanocomposites increased compared to virgin blends without HNTs. Blends of PLA/PA11 (80/20) reinforced with halloysite nanotubes (HNTs) were studied by Rashmi et al. [37]. The authors reported that the interface between PLA and PA11 phases is much stronger after adding 2 wt.% of HNTs compared to the virgin blend. However, beyond this critical loading rate, a discontinuous fibrillar structure comparable to that of virgin blends is observed. A study by Kennouche et al. [38] performed on PHBV/PBS blends containing both HNTs and a PHBV-*g*-MA compatibilizer revealed the occurrence of a synergistic effect between the compatibilizer and the nanofiller, which results in a significant increase in the thermal and mechanical properties of PHBV/PBS blends. From the short discussion above, the addition of nanofillers into a polymer blend matrix may act in two ways: first, they promote a transfer of stresses from the filler to the matrix, and secondly, they help to minimize phase separation and improve their interfacial adhesion. Although some papers on the compatibility of blends based on PLA/PHBHHx are reported in literature [39,40], to the best of our knowledge, there are not yet any published works on the ternary blend of PLA/PHBHHX/HNT nanocomposites. Therefore, the objective of this paper is to study the combined effect of HNTs as reinforcements and PLA-*g*-MA as a compatibilizer on morphology and properties of a PLA/PHBHHx (75/25) blend prepared via melt compounding. The roles and performances of HNTs and PLA-*g*-MA in improving the morphology as well as the physical properties in terms of mechanical, thermal, viscoelastic, and flammability properties of the polymer blends are reported and commented.

## 2. Materials and Methods

### 2.1. Materials

PLA in a pellet form was supplied by NatureWorks© (Plymouth, MI, USA) under IngeoTM, 2003D extrusion grade. The most important physical properties of the polymer are as follows: D-isomer content (optical purity) = 4.3%; density = 1.24 gcm^−3^; glass transition temperature (T_g_) = 60 °C; and melting temperature (T_m_) = 165 °C. PHBHHx in a pellet form was kindly supplied by Kaneka Corporation (Tokyo, Japan) as Aonilex X151 grade with a molar ratio of 3HHx = 10.5 mol. The polymer had the following physical characteristics: density = 1.19 gcm^−3^; T_g_ = −0.2 °C; and T_m_ = 130 °C. Halloysite nanotubes were supplied by the Algerian Kaolin Company (Soalka, Jijel, Algeria) and were collected from the Djebel Debbagh mining area in northeastern Algeria (Guelma). Halloysite nanotubes (HNTs) have an average internal diameter of 10 to 30 nm, an external diameter of 30 to 50 nm, a length of approximately 0.1 to 2 microns, a density of 2.1 g cm^−3^, and a specific surface area of 51.4 m^2^/g [38,41]. Before use, the raw clay was ground to powder using a Retsch ZM 200 rotor mill (Fisher Scientific, Loughborough, UK). After sieving, microparticles with an average diameter of 25 µm were selected. Maleic anhydride (MA) and dicumyl peroxide (DCP) were supplied by Sigma-Aldrich and used as received.

### 2.2. Sample Preparation

#### 2.2.1. Preparation via Reactive Extrusion of PLA-*g*-MA

Reactive extrusion was used to graft maleic anhydride (MA) onto PLA chains. This method requires the optimization of various parameters including the content ratio of MA and DCP and the processing parameters, i.e., temperature and screw speed. According to the data in the literature [42,43], the optimized conditions are as follows: 97 wt.% of PLA, maleic ratio of DCP = 0.4 wt.%, and 3 wt.% for MA. The ratio was set at that of PLA. Prior to processing, PLA was dried overnight at 70 °C. The melt mixing of PLA, DCP, and MA was performed using a twin-screw extruder (DSM Xplore, Geleen, The Netherlands). PLA and DCP were first mixed manually before they were introduced into a micro-compounder at 180 °C and 50 rpm for 3 min. Finally, the grafting reaction was completed by adding MA, and the process was kept running for 4 min.

#### 2.2.2. Sample Preparation of PLA/PHBHHx Blends with HNTs and PLA-*g*-AM

Prior to melt blending, PLA, PHBHHx, and PLA-*g*-MA were dried in an oven overnight at 70 °C, while HNTs were dried at 105 °C for 12 h to prevent moisture uptake and any further polymer hydrolysis. Melt compounding of samples based on PLA, PHBHHx, and HNTs was carried out in a Thermo Haake Rheomix 600 internal mixer equipped with counter-rotating rotors operating at 50 rpm at 180 °C for 7 min. The formulation codes and compositions are given in Table 1. PLA-*g*-MA was added at mid-processing time to ensure that the PLA melted. The resulting materials were ground and dried at 80 °C for 8 h. The next processing step was compression molding using the Model CARVER 3856 CE hydraulic press (Carver Inc., Wabash, IN, USA) to prepare standardized specimens for tensile and impact tests. For each form of characterization, the samples were put in a multi-adapter and covered with Teflon film heated to 180 °C between two metal plates. In detail, the polymeric materials were first pressed at low pressure for 2 min (3 degassing cycles), allowing for the material to melt and flow homogeneously. This was then followed by a high-pressure cycle at 10 bars for 4 min, and finally, the whole assembly was cooled to room temperature. Neat PLA and PHBHHx were subjected to the same experimental conditions.

### 2.3. Characterization Techniques

#### 2.3.1. Chemical Titration

To determine the amount of maleic anhydride (MA) that reacted with PLA, the titration method was used [44,45]. First, 2.5 g of the grafted samples were dissolved in 40 mL of chloroform and 1.5 mL of a 1M HCl solution at room temperature to hydrolyze the anhydride function to carboxylic acid function. Then, the supernatant was precipitated with 400 mL of ethanol, and filtered and dried under vacuum at 80 °C for 24 h. Finally, 0.4 g of the dried purified PLA-*g*-MA was then dissolved in 20 mL of chloroform. The resulting solution was titrated with KOH (potassium hydroxide) (0.06M) in methanol in the presence of phenolphthalein as the color indicator. PLA-*g*-MA was totally soluble, and no precipitate was formed during titration.

Three trials were conducted to ensure the reliability of the results. MA content was calculated using Equation (1) [45]:

(1) $$\%MA\left(\%wt\right)=\frac{N_{KOH}\times V_{KOH}}{2Wp}\times 98.06\times 100$$

where V_KOH_ is the volume of KOH; N_KOH_ is the normality of KOH; 98.06 is the molecular weight of maleic anhydride; and W_P_ is the tested weight.

#### 2.3.2. Fourier-Transform Infrared Spectroscopy (FT-IR)

Grafting of maleic anhydride on PLA was studied by Fourier-transform infrared spectroscopy (FT-IR) using a Bruker spectrometer (Bruker, Billerica, MA, USA) in ATR mode to identify the functional groups of PLA and PLA-*g*-MA. The FT-IR spectra were recorded in the 400–4000 cm^−1^ region with 32 scans at a resolution of 4 cm^−1^. This analysis was carried out directly on PLA and PLA-*g*-MA films.

#### 2.3.3. Scanning Electron Microscopy (SEM)

The fracture surface morphology of the samples was investigated using scanning electron microscopy with energy-dispersive X-ray spectroscopy (SEM/EDX) on a Quanta 250 FEI at a 10 kV acceleration voltage. The samples were cryogenically fractured in the transverse direction and coated with conductive carbon before analysis.

#### 2.3.4. Thermogravimetric Analysis (TGA)

Thermogravimetric analysis (TGA) measurements were performed on a Perkin Elmer STA 8000 thermal analyzer. Each sample at about 14 mg was subjected to a dynamic heating program from 30 to 600 °C at a heating rate of 10 °C/min under a nitrogen atmosphere. The reported values on the degradation temperatures represent an average of 3 tests.

#### 2.3.5. Mechanical Properties

Tensile measurements were performed using a Zwick/Z010 universal tensile testing machine (ZwickRoell, Ulm, Germany). Dumbbell-shaped specimens (ISO 527-2:2012 type 1BA standards [46]) were tested at a cross-head speed of 10 mm·min^−1^ at 23 °C. The values of Young’s modulus, elongation at break, and tensile strength represent an average of at least five measurements. Impact strength was determined using Izod impact tester (Ceast Resil Impactor Junior, Ceast 6546/000, Torino, Italy) with an energy of 7.5 joules according to ASTM D256-10(2018) [47]. Notched impact specimens were prepared according to standard dimensions (63 × 12.7 × 2 mm). At least five specimens were tested, and the average values were reported.

#### 2.3.6. Differential Scanning Calorimetry (DSC)

Differential scanning calorimetry (DSC) experiments were performed using a Perkin-Elmer Diamond DSC (PerkinElmer, Waltham, MA, USA). Samples of approximately 10 mg were packed in aluminum pans using a Perkin Elmer STA 8000 thermal analyzer. The samples were first heated from 30 to 200 °C at a heating rate of 10 °C/min. Next, the samples were cooled to 30 °C at a cooling rate of 10 °C/min; then, they were heated again to 200 °C at the same heating rate. The crystallinity index (Xc) of the samples is determined according to Equation (2) [48,49]:

(2) $$Xc(\%)=\frac{\Delta Hm}{\Delta H{}^{\circ}m*w}\times 100\;$$

where ΔH_m_ represents the melting enthalpy obtained from the heating scan, ΔH°_m_ is the melting enthalpy of a 100% crystalline PLA (93.7 J/g [50], and PHBHHx (115 J/g) [51] and w represent the weight fraction of the polymer matrix in the blend and nanocomposites.

#### 2.3.7. Pyrolysis Combustion Flow Calorimeter (PCFC)

To investigate flammability and combustibility, calorimetric measurements using pyrolysis-combustion flow calorimeter (PCFC) were carried out (Fire Testing Technology (FTT), East Grinstead, UK). A total of 2–4 mg of samples was subjected to an initial heating at 1 °C/s to 450 °C under a nitrogen atmosphere. Then, the pyrolysis products were directly transferred to a combustion chamber at 900 °C in a N_2_/O_2_ (80/20) atmosphere to carry out a complete combustion. The heat release rate (HRR) was calculated according to Huggett relation based on oxygen consumption (1 kg of oxygen consumed corresponds to 13.1 MJ of heat released) [52].

#### 2.3.8. Dynamic Mechanical Analysis (DMA)

Viscoelastic properties of PLA, PHBHHx, and their blends were determined using a dynamic mechanical analyzer (DMA 50N, 01dB-Metravib, Acoem, Limonest, France). The analysis was measured in tensile mode at a constant frequency of 1 Hz, and the displacement was fixed at 10 μm from −20 to 200 °C at 5 °C/min. The samples, in the form of bars with dimensions of 30 × 4 × 2 mm, were cut according to ISO 527-2 1BA dumbbells.

## 3. Results and Discussion

### 3.1. FT-IR Analysis of PLA-g-MA Spectra

**Figure 1:**
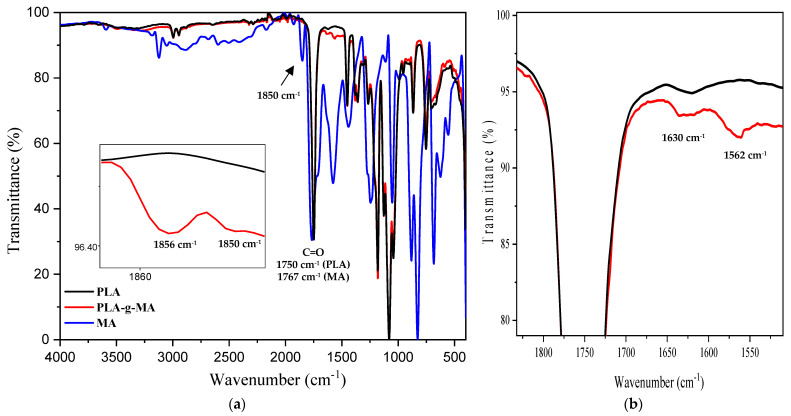
Normalized FT-IR spectra of neat PLA, PLA-*g*-MA, and MA recorded in the following regions: (**a**) 4000–400 cm^−1^; (**b**) 1830–1500 cm^−1^.

FT-IR spectroscopy was used to observe any changes occurring in the chemical structure of PLA after the grafting reaction with MA in the presence of DCP, which was used as the radical initiator. Figure 1a,b show the FT-IR spectra of neat PLA, maleic anhydride (MA), and PLA-*g*-MA recorded in transmittance mode in the domains of 4000–400 and 1830–1500 cm^−1^, respectively.

In Figure 1a, the FT-IR spectrum of pure maleic anhydride displays the presence of two characteristic absorption bands located at λ_max_ = 1767 cm^−1^ of a relatively high intensity, while the other one of a less intensity appears at λ_max_ = 1850 cm^−1^. These two absorption bands are due to the symmetrical and asymmetrical stretching of carbonyl groups (C=O), respectively. Typical absorption bands in the FT-IR spectrum of the clean PLA are clearly visible at 1780–1700, 1500–1320, and 1300–1000 cm^−1^, and are correlated with C=O stretching, CH and CH_3_ bending, and C-O stretching, respectively. This is consistent with the data found in the literature [53].

The FT-IR spectrum of PLA-*g*-MA shows a broadening absorption band in the range of 1886-1850 cm^−1^, which is not observed for neat PLA, as illustrated in Figure 1a. According to the literature [42,54], this is due to the asymmetric C=O stretching in the succinic anhydride ring. Moreover, new shoulders are also apparent in the FT-IR spectrum of PLA-*g*-MA at about 1630 and 1562 cm^−1^, which are not observed for neat PLA, as clearly shown in Figure 1b. The shoulders are assigned to the C=O and C=C stretching of the anhydride group, respectively, which is in agreement with the data in the literature [42,55]. In view of these results, it is clear that the reactive carbonyl groups of the anhydrides are present in PLA-*g*-MA, suggesting that the MA molecules are grafted onto PLA backbones, converting MA into saturated anhydride (succinic anhydride).

The amount of grafted MA is determined with the titration method using Equation (1), and the value obtained is approximately 0.62% MA unit grafted onto PLA. The functionalization of PLA should result in high reactivity with a wide range of polymer chains and biological macromolecules [56].

### 3.2. Morphology

**Figure 2:**
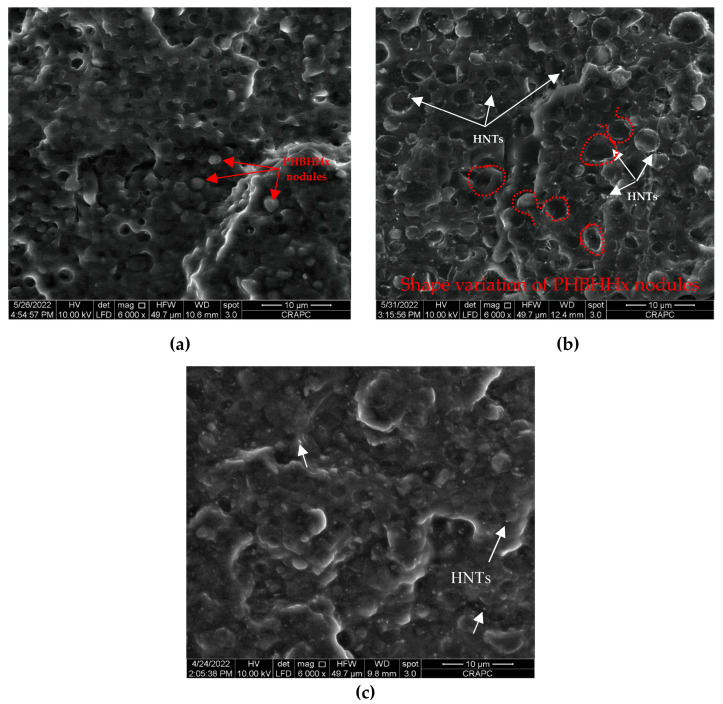
SEM micrographs of (**a**) description of what is contained in the first panel; (**b**) PLA/PHBHHx/HNT (75/25) with 5 wt.% of HNTs; (**c**) PLA/PHBHHx/HNTs (75/25) with 5 wt.% of HNTs and 10 wt.% of PLA-*g*-MA.

A morphological investigation is of crucial importance to evaluate the effect of HNTs and PLA-*g*-MA compatibilizer, either separately or combined, on the microstructure of the PLA/PHBHHx (75/25) blend. Accordingly, the SEM micrographs of the fracture surface of the PLA/PHBHHx blend with 5 wt.% of halloysite nanotubes with and without 10 wt.% of PLA-*g*-MA are shown in Figure 2a,b,c, respectively.

Figure 2a shows the SEM micrograph of the fracture surface of a virgin blend of PLA/PHBHHx. It is clearly observed to have a fine nodular biphasic morphology, where PHBHHx is uniformly dispersed in the PLA matrix. However, some microvoids appear on the surface due to the removal of PHBHHx nodules, indicating a weak interfacial adhesion between PLA and PHBHHx. Figure 2b,c show the SEM micrographs of the fracture surface of the PLA/PHBHHx blend with 5 wt.% of HNTs without and with PLA-*g*-MA, respectively. In Figure 2b, the SEM observations show a slight deformation in the shape of the PHBHHx nodules compared to the blend (Figure 1a). As can be seen in Figure 2c, the addition of PLA-*g*-MA to the PLA/PHBHHx/HNT ternary blend provides a good dispersion of HNT particles in the polymer matrix, as we also observe the disappearance of micronic aggregates of the mineral filler. The SEM micrographs clearly show that the addition of PLA-*g*-MA to the PLA/PHBHHx/HNT nanocomposite significantly improves the morphology of the ternary blend. The size of the nodules is drastically reduced, and the fracture surface appears to be less sharp, indicating a more homogeneous morphology than the non-compatibilized one.

According to the literature [23,25], the position of nanoparticles in polymer blends has a significant impact on the final structure and properties of nanocomposites.

**Figure 3:**
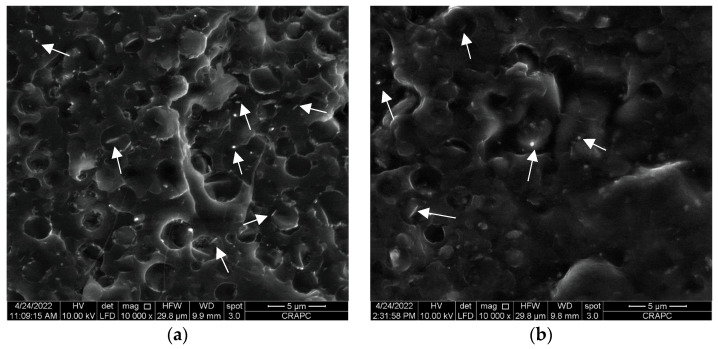
SEM micrographs of (**a**) PLA/PHBHHx/HNTs (75/25) with 5 wt.% of HNTs; (**b**) PLA/PHBHHx/HNTs (75/25) with 5 wt.% of HNTs and 10 wt.% of PLA-*g*-MA. (The white arrows point to halloysite nanotubes).

For this purpose, the position of halloysite nanotubes (HNTs) in the PLA/PHBHHx blend in the absence and presence of a PLA-*g*-MA compatibilizer is examined more closely at a higher magnification, i.e., 10,000×, as shown in Figure 3a,b, respectively.

It is observed in Figure 3a that HNTs, which clearly appear on the fracture surface of the polymer blend, are preferentially located in the PHBHHx phase and remain trapped. This may explain the slight increase in the size and deformation of the PHBHHx nodules observed in Figure 2b. Moreover, a part of the halloysite nanotubes is also located at the interface between PLA and PHBHHx. This heterogeneous distribution of HNTs in the blend could be one of the main reasons for the limited development of the tensile. After adding PLA-*g*-MA to the blend (see Figure 3b), we observe that the HNTs seem covered by a uniform polymer layer. Furthermore, a better cohesion at the interface between PLA and PHBHHx is clearly demonstrated in Figure 2c and Figure 3b. Indeed, a smoother and a more homogeneous transition between the PLA and PHBHHx regions can be observed. The boundaries between these two polymers appear to be less distinct and more integrated, attesting a closer interaction between them. This accumulated cohesion also translates into a more uniform texture at the interface, where the two polymers seem to gradually melt into each other. These SEM images demonstrate the effectiveness of the compatibilizer in reducing interfacial tension and improving adhesion between the blend components.

### 3.3. Mechanical Properties

Table 2 summarizes the main mechanical data, i.e., the tensile strength, Young’s modulus, elongation at break, and Izod impact resistance, of neat PLA, neat PHBHHx, PLA/PHBHHx, and PLA/PHBHHx/HNT blends with and without a compatibilizer. From Table 2, it can be seen that the addition of 25 wt.% of PHBHHx to the PLA matrix results in an increase in the elongation at break, passing from 4.2% for neat PLA to 12.7% for the polymer blend. A similar trend is also observed with impact resistance, which increases from 4.5 kJ/m^2^ for neat PLA to almost 13 kJ/m^2^ for the blend, but to the detriment of the stiffness and tensile strength. This is well expected given the flexible nature of PHBHHx. Based on the law of mixtures, it clearly appears that the PLA/PHBHHx (75/25) blend is characterized by intermediate tensile properties between stiffness and ductility. This behavior has already been reported by Zhao et al. [16] and Lim et al. [17] in their studies on PLA/PHBHHx blends prepared via melt blending. This is due to the low amount of PHBHHx that is incorporated in the blend (<20 wt.%), which is easy to disperse finely and homogeneously in the PLA matrix.

As reported in the literature [57,58], when nanoparticles are uniformly dispersed in the polymer matrix, they may act as reinforcing agents for polymeric materials. Moreover, when the nanoparticles are located at the polymer–polymer interface, they can also act as compatibilizers [22]. According to Table 2, the incorporation of 5 wt.% of halloysite nanotubes (HNTs) to the PLA/PHBHHx blend results in a slight decrease in the value of tensile strength compared to the virgin one, while the Young’s modulus slightly increases. By correlating the tensile data with the morphological observations presented in Figure 2b and Figure 3a, it can be assumed that the decrease in tensile strength observed in the PLA/PHBHHx/HNT nanocomposite is due to a limited adhesion between the HNT particles and the PLA matrix at the polymer blend interface.

Indeed, the hydrophilic nature of halloysite nanotubes prevents any compatibility with the highly hydrophobic polymer blends. In addition, the SEM images indicate a relatively low content of HNTs in the PLA phase. This non-uniform distribution of HNTs in the nanocomposite may weaken the interfacial adhesion and lead to a reduction in the tensile strength [59]. Interestingly, the elongation at break of the nanocomposite remains almost constant, meaning that the HNTs do not affect the materiel’s ductility. Another important result is that the impact resistance increases by 22% compared to the virgin PLA/PHBHHx blend. This increase can be attributed to the dissipation of impact energy through the incorporation of HNTs acting as energy absorbers, which helps to improve the materials’ resistance to fracture [60]. Similar observations were also reported in the literature [61,62].

**Figure 4:**
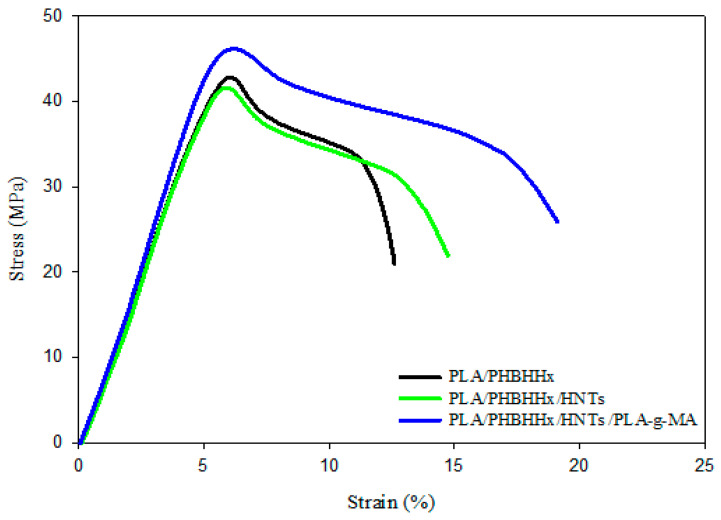
Stress–strain curves for PLA/PHBHHx and PLA/PHBHHx/HNTs ternary blend with compatibilizer.

The stress–strain plots in Figure 4 clearly show the effectiveness of combining HNTs and PLA-*g*-MA to improve the strain resistance of the PLA/PHBHHx blend. Indeed, the percentage increases in the tensile strength, elongation at break, and impact strength are approximately 12, 25, and 50% compared with the blend filled with halloysite nanotubes, which is still much higher than the unfilled PLA/PHBHHx blend. The data in Table 2 also indicate an increase in the elastic modulus by almost 7% compared with that of the non-compatibilized nanocomposite blend. Improved tensile properties as well as impact strength may be attributed to the efficacy of the compatibilizer to enhance the interfacial adhesion between the blend components of the PLA/PHBHHx/HNT nanocomposite. The occurrence of some interactions between the anhydride functional group of PLA-*g*-MA with the hydroxyl groups of HNTs and PHBHHx chains may be responsible for promoting the interfacial adhesion between them, which is in good agreement with the morphological observations (Figure 2c and Figure 3b).

### 3.4. Thermogravimetric Analysis (TGA)

**Figure 5:**
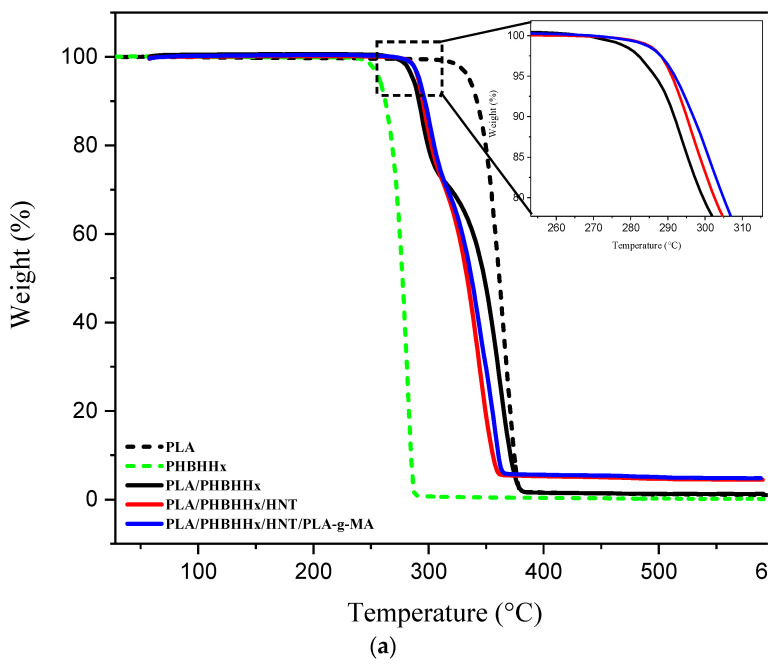

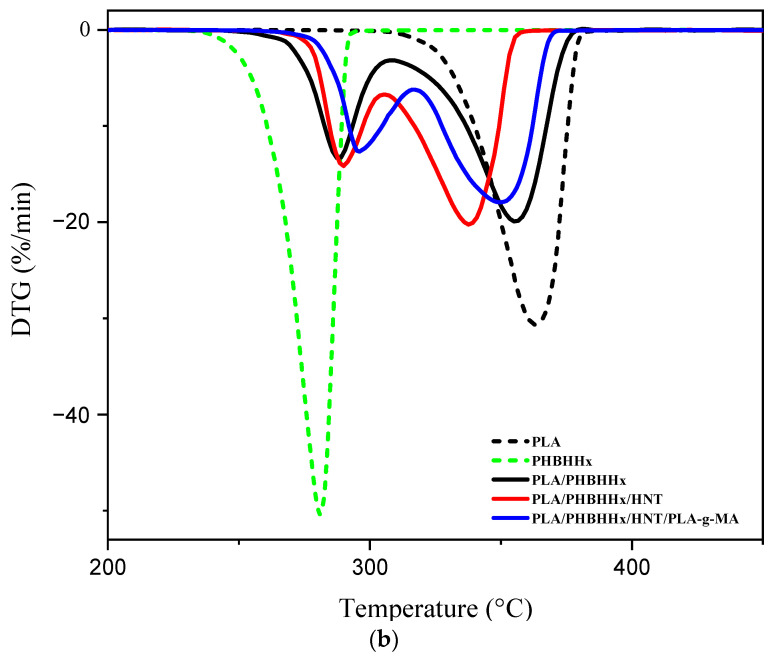
TGA (**a**) and DTG (**b**) curves of the different samples.

The thermal stability of PLA, PHBHHx, the PLA/PHBHHx blend, and PLA/PHBHHx/HNT in the absence and presence of a compatibilizer was investigated via TGA. The corresponding TGA and DTG thermograms are shown in Figure 5a,b, respectively. Further, Table 3 summarizes the main data.

From Figure 5a, PLA and PHBHHx exhibit a one-step degradation process starting at almost 257 °C for PHBHHx and 335 °C for PLA, which is consistent with the data in the literature [16]. A similar trend is also observed for the DTG curves, as shown in Figure 5b. For the PLA/PHBHHx blend, there are two thermal degradation steps. The first step is due to PHBHHx degradation, while the second one is relative to that of PLA. In Table 3, the TGA data indicate that for the PLA/PHBHHx blend, the temperature value at 5 wt.% mass loss (T_5_) is almost 285 °C and falls between that of PLA, which is roughly 335 °C, and PHBHHx, which is about 256 °C. The increase in T_5_ may be due to a good dispersion of halloysite nanotubes in the polymer blend. Moreover, according to some authors [63], the tubular structure of HNTs could trap volatile products that are released during thermal degradation in the lumen of HNTs, thus imparting more thermal stability. For the nanocomposite blend, the thermal stability depends not only on the good dispersion of the nanofiller, but also on its location in the polymer phases [27]. The thermal stability was found to improve after the addition of HNT and PLA-*g*-MA. The T_5_ value of the virgin PLA/PHBHHx blend increased from 285 °C to about 290 and 291 °C in the presence of only halloysite nanotubes and in the presence of both HNTs and PLA-*g*-MA, respectively.

However, for the second stage of degradation (more than 27% mass loss), the curve shifted to lower temperatures. This unexpected observation can be attributed to a hydrolysis phenomenon, resulting from several factors including the dispersion state of HNTs in the PLA matrix, and agglomerated halloysite nanotubes, which are also able to create localized regions in a higher water concentration, speeding up hydrolysis in these zones. It is also plausible that the products resulting from the first degradation of PHBHHx plays an active role in the PLA degradation process. Polymeric degradation often leads to the formation of low-molecular-weight compounds, such as oligomers, monomers, and smaller fragments. These degradation products may possess functional groups or chemical motifs that are sensitive to hydrolysis. On the other hand, Prasad et al. [64] and Yuanyuan et al. [65] reported that the limited thermal stability improvement for PLA/HNTs could be due to the presence of voids at filler–PLA interfaces.

Figure 5b shows the DTG curves for the samples, where the maximum weight loss temperature (Tmax) for each sample is clearly highlighted. PLA has a higher Tmax value (363 °C) than PHBHHx (281 °C). The blend and nanocomposites exhibit two temperature peaks (Tmax_1_ and Tmax_2_), which correspond to PHBHHx and PLA, respectively. The Tmax of PLA in the blend shifts to lower temperatures, whereas that of PHBHHx (Tmax_2_) slightly increases. For the nanocomposite reinforced with halloysite nanotubes, Tmax_1_ slightly increases, while Tmax_2_ decreases. For the nanocomposite compatibilized with PLA-*g*-MA, there is a slight increase in the Tmax_1_ (+5 °C) and a significant increase in the Tmax_2_ (+11 °C) compared with the Tmax of the nanocomposite before compatibilization.

An improvement in the Tmax of PLA in the presence of PLA-*g*-MA may be due to an improvement in the PLA-HNT interfacial adhesion. The hydroxyl groups of HNTs likely react with MA groups to form covalent bonds between the filler and PLA. This reaction reduces the reactivity of hydroxyl groups for other chemical reactions, including hydrolysis mechanisms.

In order to estimate the possible interactions between the components of the blend, the mass loss value was calculated from the separate data for each component as the total of the individual contributions, assuming that there were no interactions. The theoretical TGA curve for the case of a binary blend was built using Equation (3) according to [38,66]. This can be extended for multicomponent blends.

**Figure 6:**
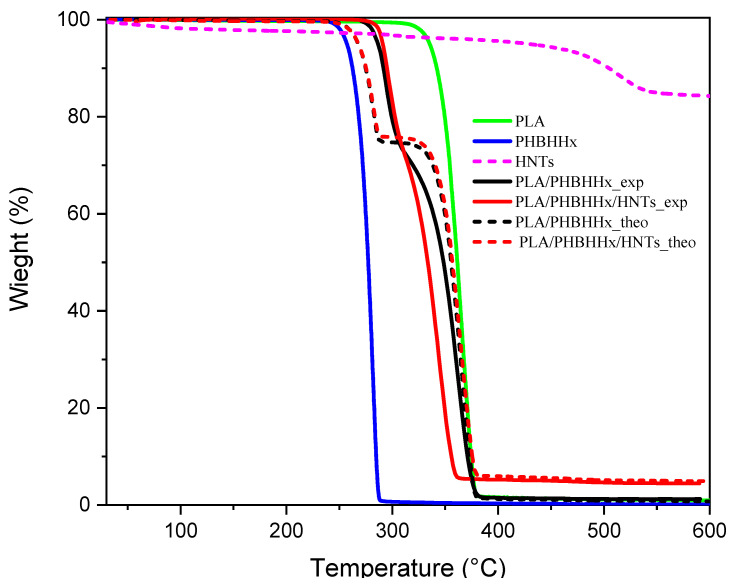
Theoretical and experimental mass loss curves; continuous lines show the effect observed experimentally, and the dashed line stands for the theoretical curves.

W**_blend_** = f_A_W_A_ + f_B_W_B_ + … + fnWn, (3)

where W is the mass loss; fis the polymer weight fraction in the blend; and A and B are the constituents of the blend. As indicated in reference [67], when the theoretical and experimental mass loss curves are identical, this indicates the absence of interactions between the components (sum of contributions). However, when the experimental and theoretical curves are different, the combined effect of the components is higher than the theoretical one (the combined effect of the components is more than the sum of their individual contributions). Figure 6 shows both the experimental and theoretical TGA curves of the PLA/PHBHHx binary blend and the PLA/PHBHHx/HNT ternary blend.

As shown in Figure 6, the theoretical and experimental curves of the PLA/PHBHHx binary blend and the PLA/PHBHHx/HNT blend show different profiles, while the amount of final residue at 600 °C remains the same. The experimental curves for the binary and ternary blends show a positive deviation in the first stage due to PHBHHx degradation, indicating the presence of interactions between the components, whereas in the second degradation stage related to PLA, a negative deviation of the experimental curves of PLA/PHBHHx/HNT is noted, suggesting that the hydroxyl groups in the halloysite nanotubes (HNTs) can catalyze PLA hydrolysis by enhancing water absorption and reducing the activation energy of the hydrolysis reaction. This leads to accelerated PLA degradation in the presence of HNTs. The products resulting from the first degradation also affect this negative deviation.

### 3.5. Differential Scanning Calorimetry (DSC)

**Figure 7:**
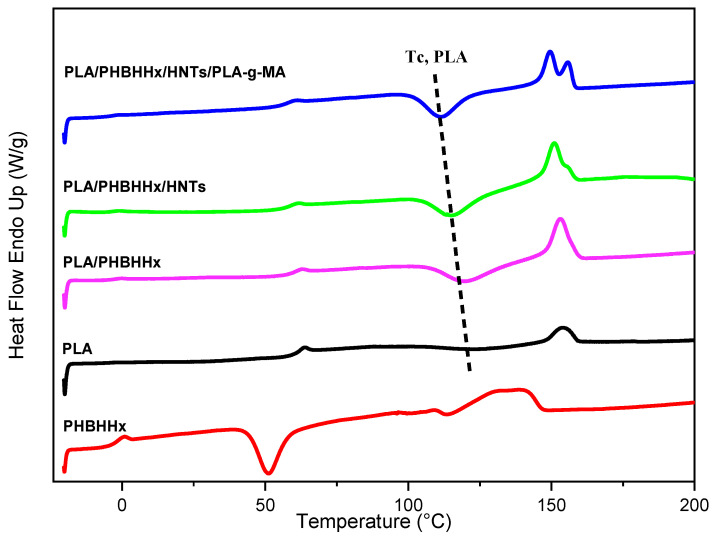
DSC thermograms of samples recorded at the 2nd heating.

Figure 7 shows the melting behaviors, determined via DSC, of PLA, PHBHHx, the PLA/PHBHHx blend, and PLA/PHBHHx/HNT with and without the compatibilizer. Moreover, the main DSC data are shown in Table 4. The DSC thermograms of the samples shown in Figure 7 display semi-crystalline polymer structures. The presence of 25 wt.% of PHBHHx in the blend increases the crystallinity rate and the area of the melting peak of PLA due to the promotion of the cold crystallization of PLA. As shown in Figure 7 and Table 4, the incorporation of halloysite nanotubes has no significant effect on the crystallinity and melting properties of the PLA/PHBHHx blend. The melting temperature and enthalpy of fusion are both similar to those of the neat blend.

A comparison between the DSC thermograms of neat PLA/PHBHHx and those of the nanocomposite with PLA-*g*-MA shows a significant variation in the crystallization and melting temperature of PLA. It is observed in Figure 7 that the cold crystallization peak shifts to lower temperatures, indicating an enhancement of the PLA chains’ mobility, whereas the crystallization enthalpy increases (Table 4). Further, two distinct temperature peaks are noted at about 151 and 155 °C.

The presence of double/multiple melting peaks is a frequently observed phenomenon in semi-crystalline aliphatic polyesters, which is due to the presence of melting and recrystallization processes. During heating, PLA with imperfect crystals melts first, followed by the remelting of PLA with more perfect crystals [68]. This phenomenon was reported by several authors [53,54,69].

### 3.6. Flammability Properties (PCFC)

**Figure 8:**
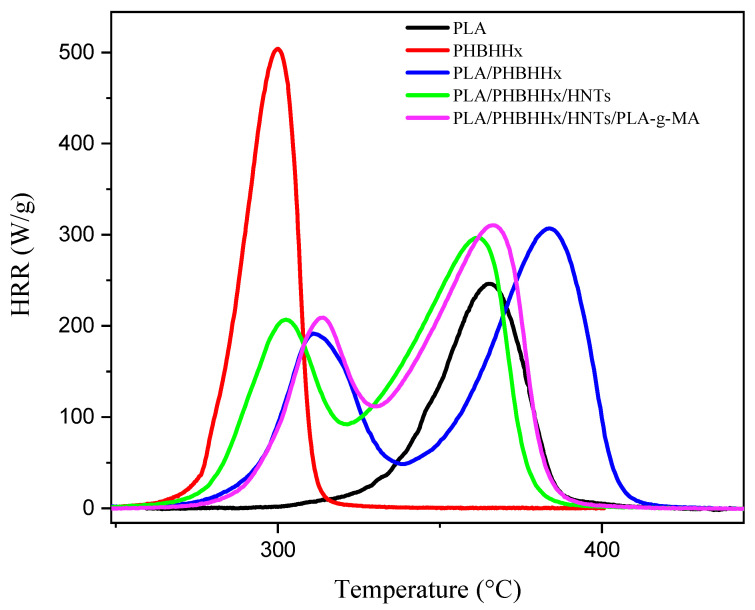
Heat release rate (HRR) curves vs. the temperature of simples.

PCFC was used to assess the sample’s flammability behavior. The temperature-dependent heat curves are shown in Figure 8, while Table 5 summarizes the main results of the test.

The curves of the peak heat release rate (pHRR) of the neat PHBHHx and neat PLA show that the two polymers have different combustion behaviors. The total heat release (THR) values for PLA and PHBHHx are 16.5 and 21.9 kJ/g, respectively, indicating that PHBHHx releases more heat during combustion than PLA. The difference in THR can be attributed to several factors such as differences in the chemical composition, molecular structure, and thermal properties of the polymers. For example, the C/O ratio and -CH_2_ content in the chemical structure of PHBHHx are higher than those of PLA. This chemical composition may contribute to the greater combustibility of PHBHHx compared with PLA. Two degradation phases are detected in the flammability behavior of the polymers, similar to what the TGA reported. The heat release rate (HRR) curves of the PLA/PHBHHx and PLA/PHBHHx/HNT blends reveal two unique peaks, which correspond to PHBHHx and PLA flammability. The presence of halloysite nanotubes (HNTs) reduces the temperature heat release rate (TpHRR) of PHBHHx and PLA compared to the neat blend. The TpHRR of PHBHHx shows a slight decrease, while the TpHRR of PLA decreases significantly by around 22 °C. This result suggests the occurrence of interactions between HNTs and the polymers, particularly PLA, since the incorporation of HNTs seems to promote hydrolysis and thermal decomposition reactions, leading to a faster release of flammable compounds. This is in a good agreement with the TGA results.

The incorporation of PLA-*g*-MA results in a slight increase in the TpHRR for both PLA and PHBHHx in the nanocomposite blend, suggesting a beneficial effect of PLA-*g*-MA on the thermal behavior of the blend.

### 3.7. Determining Viscoelastic Properties via DMA

**Figure 9:**
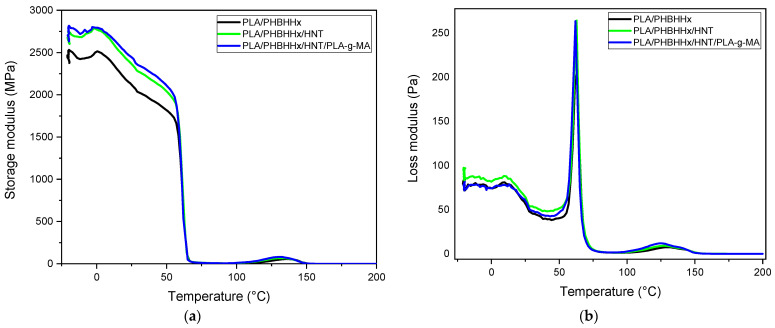

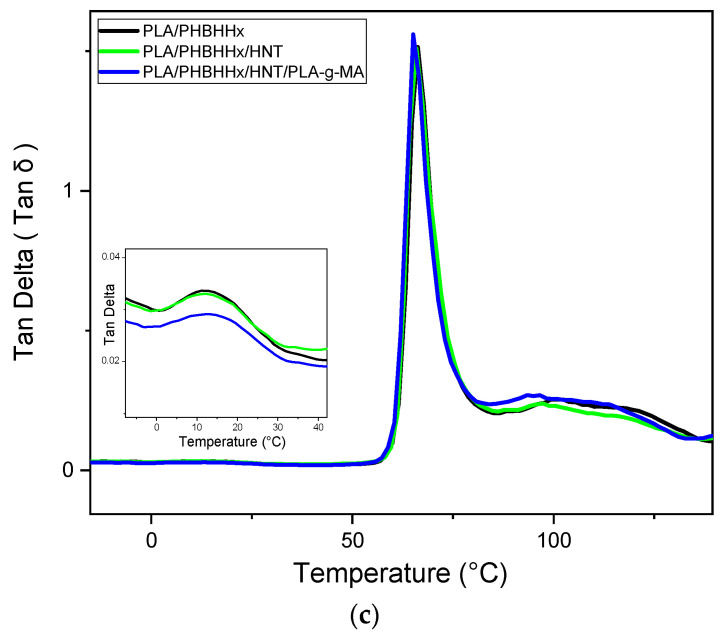
Evolution of (**a**) dynamic storage modulus; (**b**) loss modulus; and (**c**) tan (δ).

DMA was performed on the samples based on the PLA/PHBHHx blend, PLA/PHBHHx/HNT, and the PLA/PHBHHx/HNTs/PLA-*g*-MA nanocomposite. The storage modulus (E’) and tan δ curves are presented in Figure 9. It is observed that the E’ of PLA/PHBHHx (with and without HNTs and PLA-*g*-MA) follows a multi-step chain relaxation process. At a temperature below the glass transition of PHBHHx, the E’ exhibits a relatively stable plateau, although a slight disturbance in the E’ values is observed. As the temperature increases, the E’ of PLA gradually decreases. Figure 9a clearly shows that the addition of HNTs and PLA-*g*-MA to the blend results in a higher storage modulus (E’) compared to the blend over the entire temperature range up to the transition temperature of PLA. This shows the reinforcing effect of halloysite nanotubes and the enhanced dispersion of HNTs in the blend matrix due to the compatibilizer.

The temperature dependence of the loss moduli (E″) of the PLA/PHBHHx blend, PLA/PHBHHx/HNT, and the PLA/PHBHHx/HNTs/PLA-*g*-MA nanocomposites is displayed in Figure 9b. The relaxation α associated with the glass transition temperature (Tg) of the samples coincides with the maximum temperature of the loss modulus shown [70,71]. All curves show two peaks at approximately Tg related to the α relaxation of PHBHHx and PLA. This is consistent with the DSC data. Furthermore, there is no change in the shape of the tan δ curves for PLA/PHBHHx/HNT and the PLA/PHBHHx/HNTs/PLA-*g*-MA nanocomposites compared with that of the binary PLA/PHBHHx (75/25) blend. Figure 9c illustrates the curves of tan (δ) of the blend samples. The addition of HNTs and PLA-*g*-MA has no significant effect on tan (δ).

The absence of significant differences in the results of the dynamic mechanical analysis with the presence and absence of PLA-*g*-MA suggests that no substantial cross-linking or grafting reaction has occurred between PLA and PHBHHx. It should be noted that the absence of significant changes in the presence of PLA-*g*-MA does not necessarily negate the overall improvement in the mechanical properties or compatibility achieved by other means. The effectiveness of PLA-*g*-MA as a compatibilizer and the reinforcing effect of halloysite nanotubes may contribute to the overall improvement in the mechanical performance of the nanocomposite.

## 4. Conclusions

Polymer blends reinforced with natural fillers are very important for developing new materials with enhanced properties, especially when these materials are bio-based and totally biodegradable. This research investigates the structure–property relationships of the novel ternary poly(lactic acid) (PLA)/poly(3-hydroxybutyrate-co-3-hydroxyhexanoate) (PHBHHx)/halloysite nanotubes (HNTs) nanocomposites prepared via melt blending. In order to achieve a better interfacial interaction between the polymer components, a laboratory-synthesized compatibilizer of maleic anhydride-grafted PLA (PLA-*g*-MA) produced via reactive extrusion was used. The FT-IR spectra and chemical titration confirmed the grafting of MAs onto the PLA matrix, and the amount of grafted MA reached 0.62 wt.%. Morphological, thermal, and mechanical characterizations were performed to understand the influences of HNTs and PLA-*g*-MA on PLA/PHBHHx (75/25 wt%).

From this study, the following conclusions can be drawn: The SEM observations show that the halloysite nanotubes are preferentially located in the PHBHHx nodules due to a better HNT-PHBHHx wettability. The combination of HNTs and a compatibilizer results in a homogeneous morphology with a decrease in the size of the PHBHHx nodules. The incorporation of HNTs into the PLA/PHBHHx blend results in a slight decrease in the tensile strength of the nanocomposites, while the modulus and elongation at break remain almost unchanged. However, the impact resistance increases by 22% compared to that of the neat PLA/PHBHHx blend. Furthermore, after the addition of a PLA-*g*-MA compatibilizer into PLA/PHBHHx/HNT, there is an increase in the tensile strength, elongation at break, and impact resistance by about 12, 25, and 50%, respectively, compared to the non-compatibilized ones. Further, a slight increase in the thermal stability of the compatibilized nanocomposite is also observed.

This article shows that blending biopolymers is an effective and promising way to adjust some of their properties. So, the use of halloysite nanotubes with a suitable compatibilizer such as PLA-*g*-MA is an additional approach to improve the performance properties of PLA/PHBHHx blends.

## Figures and Tables

**Table 1:** Codes and compositions of the formulations investigated.

Formulations	PLA (wt.%)	PHBHHx (wt.%)	HNTs (wt.%)	PLA-*g*-MA (wt.%)
PLA	100	/	/	/
PHBHHx	/	100	/	/
PLA/PHBHHx	75	25	/	/
PLA/PHBHHx/HNTs	71.25	23.75	5	/
PLA/PHBHHx/HNTs/PLA-*g*-MA	63.75	21.25	5	10

**Table 2:** Young’s modulus, tensile strength, elongation at break, and impact resistance values of PLA, PHBHHx, PLA/PHBHHx blend, and PLA/PHBHHx/HNTs with and without compatibilizer.

Formulations	Young’s Modulus (MPa)	Tensile Strength ((MPa)	Elongation at Break (%)	Impact Strength ((kJ/mm^2^)
PLA	3438 ± 57.4	58 ± 6.2	4.1 ± 1.4	4.5 ± 0.1
PHBHHx	840.4 ± 35.6	18.3 ± 1.2	18.6 ± 2.6	14,7 ± 1.9
PLA/PHBHHx	2117 ± 41	43.4 ± 0	12.7 ± 1.5	12.7 ± 1.9
PLA/PHBHHx/HNTs	2233 ± 88	42 ± 0	14.5 ± 1.2	20.9 ± 1.7
PLA/PHBHHx/HNTs/PLA-*g*-MA	2390 ± 75	46 ± 1	18.2 ± 2.8	31.2 ± 1.3

**Table 3:** Main values of thermal characteristics determined via TGA/ DTG.

Formulations	T_5_ (°C)	T_50_ (°C)	Tmax (°C)		Char Yield (%) at 600 °C
			PHBHHx	PLA	
PLA	335.4 ± 1.3	361.6 ± 0.6	/	363.4 ± 1.2	1.01 ± 0.09
PHBHHx	256.2 ± 1.1	277.4 ± 0.4	281.6 ± 0.6	/	0.19 ± 0.04
PLA/PHBHHx	285.2 ± 1.2	348.7 ± 0.8	287.2 ± 0.7	355.3 ± 0.8	355.3 ± 0.8
PLA/PHBHHx/HNTs	290.4 ± 0.9	333.4 ± 0.7	289.6 ± 0.9	339.2 ± 1.3	1.20 ± 0.11
PLA/PHBHHx/HNTs/PLA-*g*-MA	291.9 ± 1.6	337.4 ± 1.1	294.2 ± 1.2	350.1 ± 0.7	4.45 ± 0.13
HNTs	428.9	/	516.1 ± 1.2		84.3 ± 1.3

**Table 4:** Thermal properties of pure PLA and PHBHHx, binary PLA/PHBHHx (75/25) blend and nanocomposite comprising PLA/PHBHHx (75/25) + HNTs with and without PLA-*g*-MA as compatibilizer.

Samples	T_g_ ±0.3 (°C)	T_c_ ±0.6 (°C)	ΔHc ±0.8 (J/g)	T_m_ ±0.5 (°C)	ΔHm ±0.5 (J/g)	Xc PLA ±0.8 (%)
PLA	59.9	/	/	153.7	6.53	6.9
PHBHHx	2.86	51.2	40.4	132.6	34.8	30.2
PLA/PHBHHx	58.8	120.1	19.6	153.1	19.9	28.3
PLA/PHBHHx/HNTs	57.2	115.5	22.6	151.05	19.7	28
PLA/PHBHHx/HNTs/PLA-*g*-MA	56.4	111.7	25.3	151.2–155.9	22.6	32.1

**Table 5:** The values for pHRR and TpHRR obtained from PCFC.

Samples	pHRR PHBHHx (W/g)	p HRR PLA (W/g)	T pHRR PHBHHx (°C)	T pHRR PLA (°C)
PLA	/	246.3	/	365
PHBHHx	503.9	/	299.8	/
PLA/PHBHHx	190.4	306.7	311.2	383.8
PLA/PHBHHx/HNTs	207.7	296.6	301.8	361.4
PLA/PHBHHx/HNTs/PLA-*g*-MA	209.2	310.6	313.6	366.4

## Data Availability

Not applicable.
